# Managing diversity: Domestication and gene flow in *Stenocereus stellatus* Riccob. (Cactaceae) in Mexico

**DOI:** 10.1002/ece3.524

**Published:** 2013-04-11

**Authors:** Jennifer M Cruse-Sanders, Kathleen C Parker, Elizabeth A Friar, Daisie I Huang, Saeideh Mashayekhi, Linda M Prince, Adriana Otero-Arnaiz, Alejandro Casas

**Affiliations:** 1 Atlanta Botanical Garden1345 Piedmont Ave NE, Atlanta, GA, 30309, USA; 2 Department of Geography, University of GeorgiaAthens, GA, 30602, USA; 3 Rancho Santa Ana Botanic Garden1500 N. College Ave., Claremont, CA, 91711, USA; 4 Agricultural Specialist, USDA Foreign Agricultural Service, U.S. EmbassyMéxico, D.F, Mexico; 6 Centro de Investigaciones en Ecosistemas, Universidad Nacional Autónoma de Méxicocampus Morelia, Morelia, Michoacán, 58190, Mexico

**Keywords:** Cactus, domestication, gene flow, microsatellite, population bottleneck, *Stenocereus*

## Abstract

Microsatellite markers (N = 5) were developed for analysis of genetic variation in 15 populations of the columnar cactus *Stenocereus stellatus,* managed under traditional agriculture practices in central Mexico. Microsatellite diversity was analyzed within and among populations, between geographic regions, and among population management types to provide detailed insight into historical gene flow rates and population dynamics associated with domestication. Our results corroborate a greater diversity in populations managed by farmers compared with wild ones (*H*_E_ = 0.64 vs. 0.55), but with regional variation between populations among regions. Although farmers propagated *S. stellatus* vegetatively in home gardens to diversify their stock, asexual recruitment also occurred naturally in populations where more marginal conditions have limited sexual recruitment, resulting in lower genetic diversity. Therefore, a clear-cut relationship between the occurrence of asexual recruitment and genetic diversity was not evident. Two managed populations adjacent to towns were identified as major sources of gene movement in each sampled region, with significant migration to distant as well as nearby populations. Coupled with the absence of significant bottlenecks, this suggests a mechanism for promoting genetic diversity in managed populations through long distance gene exchange. Cultivation of *S. stellatus* in close proximity to wild populations has led to complex patterns of genetic variation across the landscape that reflects the interaction of natural and cultural processes. As molecular markers become available for nontraditional crops and novel analysis techniques allow us to detect and evaluate patterns of genetic diversity, genetic studies provide valuable insights into managing crop genetic resources into the future against a backdrop of global change. Traditional agriculture systems play an important role in maintaining genetic diversity for plant species.

## Introduction

The evolution of key characteristics in crops through domestication marks one of the most important shifts in ecology and life history for certain plant species. Descriptions of plant domestication and patterns of inheritance have been used to infer more general evolutionary processes since the early development of evolutionary theory (Darwin 1859, 1875). Newer models suggest that, early crop domestication resulted from slower processes than first hypothesized (Allaby et al. 2008; Fuller et al. 2009). These analyses suggest that artificial selection for traits important to human societies, such as retention of seeds in cereal crops, was slowed as a result of sympatry between wild and cultivated populations early in the process of domestication (Fuller et al. 2009).

Classical models of plant domestication involve artificial selection and propagation of plants with desirable characteristics, resulting in shifts in the frequency of desirable traits (Doebley 1989) and bottlenecks of genetic and phenotypic variation, as is seen within many modern domesticated species (Sauer 1972; Doebley 1992; Tanksley and McCouch 1997; Buckler et al. 2001). Most plant domestication events were assumed to happen ex situ (ie, removed from their wild habitat). However, recent study has shown that crops may have coexisted with wild relatives for up to a millennium during domestication (Tanno and Willcox 2006). Whether genetic patterns within crop species result from an initial bottleneck early in the domestication process or from protracted bottlenecks and gene flow events over many generations is still a matter of debate (Eyre-Walker et al. 1998; Ellstrand et al. 1999; Allaby et al. 2008).

Mesoamerica is an important domestication center for hundreds of plant species including crops of worldwide importance, such as maize, squash, beans, and peppers, and numerous semi-domesticated species (Harris 1967; MacNeish 1967; Harlan 1975; Whitmore and Turner 1992; Casas et al. 2007; Blancas et al. 2010). The Tehuacán Valley (TV) and the Balsas River Basin are regions of Mexico with a particularly rich and long history of human and plant interaction (MacNeish 1992) supporting an unusually high diversity of native plants used by inhabitants since its early occupation millennia ago. For instance, in a survey of ethnobotanical resources for the Tehuacán-Cuicatlán Valley, Lira et al. (2009) reported approximately 1600 regional (native and introduced) plant species useful to humans, including a significant number that showed evidence of management and domestication. Blancas et al. (2010) documented that 600 of these species receive some management type other than simple gathering, and almost 350 of the managed plant species were native to the TV.

The TV and the Balsas River Basin are among the main areas of plant domestication in Mesoamerica (MacNeish 1992; Casas et al. 2007; Zizumbo-Villarreal and Colunga-GarciaMarin 2010). Characteristics of managed plant species in these areas range from incipient domestication to advanced dependence on humans for survival and reproduction (Casas et al. 2007; Blancas et al. 2010). Accumulating evidence suggests that this results from a combination of artificial selection, genetic drift, and gene flow due to the diversity of cultural roles and life histories of plant species in this region. Understanding the population processes of different species from these regions will significantly contribute to developing theories on evolution under domestication.

**Figure 1:**
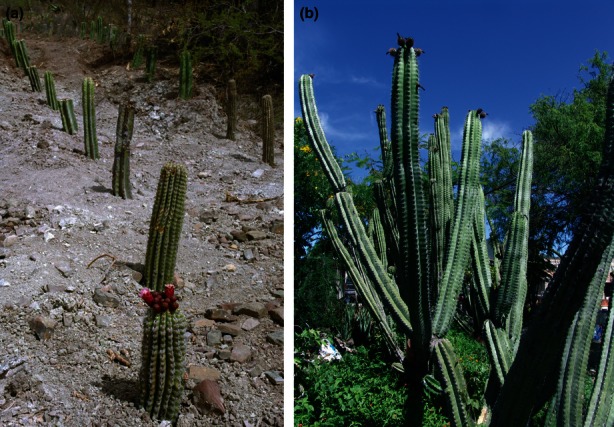
(a) Photo of clonally propagated cacti in a managed population near San Juan Raya, wild population (Puebla, Mexico) in 2007. *Stenocereus stellatus* is flowering in the foreground. (b) Mature *S. stellatus* with fruit cultivated in home garden in Chinango, Oaxaca, Mexico in 2008.

This study examines population processes resulting from management of plants in traditional agriculture systems to provide insight into both evolutionary processes involved in crop domestication and the response of crops to changing evolutionary forces in the future. Specifically, we evaluated spatial variation in genetic diversity and underlying evolutionary processes in populations of a semi-domesticated species, *Stenocereus stellatus* Riccob., throughout its natural distribution range in central Mexico. This study develops our previous research which identified three different management systems of *S. stellatus* found in wild populations, managed in situ among pastures and agroforestry fields, and cultivated in home gardens (Casas et al. 1997, 2006), to examine how diversity is maintained and transferred throughout the system. Most in situ management occurs when farmers clear land for maize cultivation and retain cacti with desirable phenotypic traits; plants with desirable phenotypes are then brought into cultivation through active planting of cactus stem segments and seeds in home gardens adjacent to dwellings (Casas et al. 1999b). Occasionally plants from home gardens may be transferred to managed in situ populations (Fig. 1; Casas et al. 1997). Ethnobotanical studies documented that plants in home gardens may be derived from local or distant populations, and that traditional farmers promote high levels of diversity (Casas et al. 1997). Casas et al. (2006) generally described spatial distribution of morphological and genetic diversity in wild and managed populations; however, analyses of genetic structure and gene flow among those populations were not reported in previous studies. Although cactus species have been used by people for 1000s of years (MacNeish 1967), this study is among the first few to report genetic analysis of traditionally cultivated cactus crops (Otero-Arnaiz et al. 2004; Caruso et al., 2010, Casas et al. 2006). Furthermore, the processes by which diversity is managed in this system have not previously been analyzed.

Given, the long history of human interaction with *S. stellatus* (Casas et al. 1999b), and its use as a trade material, it is possible that plants in home gardens are not of local origin, with consequences for the genetic structure of the species and conservation efforts, as has been found for other semi-domesticated species (Miller and Nair 2006; Dawson et al. 2008). The natural distribution of *S. stellatus* comprises portions of the neighboring semiarid regions of the Balsas River Basin (particularly La Mixteca Baja region [LMB]) and the TV, which have significant environmental differences. LMB is more humid than the TV, and such differences are relevant in understanding both natural and cultural histories of the species since seedling recruitment differs significantly according to water availability (Guillén et al. 2009, 2011; Guillén 2012).

Our analysis focused on three hypotheses:

1. If domestication in this species has resulted from in situ artificial selection of wild plants, then genetic bottlenecks and shared genotypes between geographically proximal managed in situ and cultivated populations (leading to isolation by distance and clustering of close populations) should both be evident.
2. If traditional farmers increase genetic diversity in their home gardens through cultivating multiple clones of desirable individuals (Casas et al. 2006), identical multilocus genotypes should occur more frequently than at random in these populations.
3. Finally, if farmers promote cultivated diversity through selection and gene exchange with neighboring farmers (geographically regional farmers; Casas et al. 2006), these relationships should be evident in cluster and migration analysis and we should see isolation by distance.

## Material and Methods

### Species biology and study area

*Stenocereus stellatus* is a small columnar cactus, reaching heights up to 4 m. It bears reddish flowers 4 to 5 cm in length, and the fruit, which ripens from May to September, ranges in size from 31 cm^3^ in wild populations to 58 cm^3^ under cultivation (Pimienta-Barrios and Nobel 1994; Casas et al. 1999a). The plants appear to have a self-incompatible breeding system and are pollinated by nectarivorous bats, particularly of the genus *Leptonycteris* (Casas et al. 1999b). These bats have been reported to fly up to 100 km per night, suggesting that the gene flow neighborhood size for *S. stellatus* may be quite large (Horner et al. 1998; Arias-Coyotl et al. 2006).

**Figure 2:**
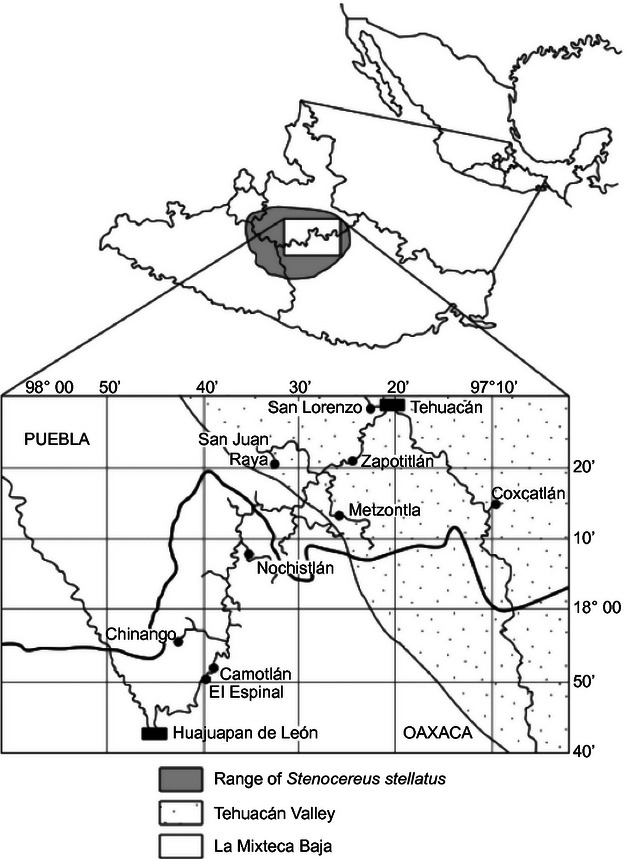
Map of collection sites for *Stenocereus stellatus*. See Table 1 for the list of populations sampled.

The range of *S. stellatus* is centered in the TV and LMB, which are part of the Papaloapan and Balsas River Basins, respectively, in south-central Mexico (Fig. 2). This area, which includes parts of the states of Puebla and Oaxaca, is one of the richest regions in the world for cactus species diversity (Bravo-Hollis 1978; Valiente-Banuet et al. 2002). Columnar cacti are predominant species within the thornscrub and tropical deciduous forests, and are common throughout the region.

Although they are adjacent regions, the TV and LMB differ physiographically, climatically, and phytogeographically (Rzedowski 1993). The relatively low elevation TV supports thornscrub and tropical deciduous forests that occupy sandstone, limestone, and volcanic substrates. LMB is a more complex mountainous region southwest of the TV, with thornscrub and tropical deciduous forests at lower elevations and pine forests at higher elevations. Mean annual precipitation in LMB is nearly twice that of the TV (600–800 mm vs. 300–500 mm, respectively; Bravo-Hollis 1978). These physical contrasts between LMB and the TV lead to differences in the frequency of sexual reproduction and recruitment in *S. stellatus* (Casas et al. 1999a). In addition, important cultural and ethnolinguistic differences exist between the two regions in terms of classification of fruit traits, management practices, and the role played by *S. stellatus* in the local economy (Casas et al. 1997).

### Field sampling

Mature individuals of *S. stellatus* were sampled from fifteen populations in TV and LMB, Mexico (Table 1). Wild populations were located outside of towns, within 2–4 km, along rocky hillsides in LMB and on alluvial fans in TV. Managed in situ populations occurred outside of town boundaries in areas relatively free of vegetation that had been cleared for pastures or agroforestry. Individuals in 100 × 50 m plots were mapped in wild and managed populations. At least five home gardens constituted each cultivated population, and all *S. stellatus* individuals were mapped in each. These included backyard gardens and agricultural fields adjacent to homes under traditional dry land farming with human energy conditions. The target random sample for genetic analysis was 30–50 floral buds; in several cases a scarcity of reproductively active individuals necessitated smaller samples. Fruits were collected from each individual for later analysis, and approximately 1 g of floral tissue was removed from each and stored in silica gel. Each sampled individual was located with a Garmin global positioning system (GPS) unit. Samples were stored at Rancho Santa Ana Botanic Garden (RSABG) for DNA extraction.

**Table 1:** Sampling localities, sample sizes, population abbreviations, and population densities for *Stenocereus stellatus*

Population	Type	Abbreviation	Sample size	Density (N/ha)
La Mixteca Baja				
Camotlán	Cultivated	CaC	9	555.2
Camotlán	Managed	CaM	42	17.2
Chinango	Cultivated	ChC	46	156.5
Chinango	Managed	ChM	41	76.0
Chinango	Wild	ChW	37	30.8
El Espinal	Cultivated	EEC	31	– a
El Espinal	Managed	EEM	40	137.5
El Espinal	Wild	EEW	16	22.0
Tehuacán Valley				
Coxcatlán	Wild	CoxW	33	396.0
Metzontla	Cultivated	MeC	47	250.0
Metzontla	Managed	MeM	10	80.0
San Juan Raya	Wild	SjrW	27	–a
San Lorenzo	Managed	SLM	43	157.0
Zapotitlán	Cultivated	ZapC	41	91.0
Zapotitlán	Wild	ZapW	42	1240.0

a Density estimates are unavailable because only reproductively active individuals were mapped in these populations.

Interviews were also conducted with home gardeners in each town and *S. stellatus* vendors in markets (N = 55) to provide information on cultivation and trade practices that might influence patterns of genetic diversity and gene movement. Interviewees included both males and females of various occupations, ages, and ethnic groups. Responses to the questions were summarized qualitatively to augment interpretation of quantitative genetic results.

### DNA extraction

A modified silica-based CTAB extraction protocol (Huang et al. 2000) was used. About, 20–30 mg of dried plant material was crushed using a BeadBeater-8 (Biospec, Bartlesville, OK) and homogenized in 1000 μL of 2 × CTAB solution. Five hundred micro liters of the resulting homogenate was then incubated with 0.4% β-mercaptoethanol and 20 mg/mL proteinase-K. The solution was then heated to 65°C to deactivate the proteinase and then incubated for 30 min at 37°C with 100 μL of 10% w/v Caylase 345 to digest polysaccharides. Undiluted ice cold chloroform was used to separate the aqueous layer. Buffers used to bind and wash DNA from silica were taken from Levison et al. (1998); adsorption buffer: 4 mol/L guanidine hydrochloride in 0.75 mol/L potassium acetate solution; wash buffer: 50% ethanol, 30% 1 × TE, 20% 1 mol/L NaCl solution. The aqueous layer was added to a tube containing hydrated silica and 500 μL of adsorption buffer. The mixture was shaken for 5 min to bind DNA in the presence of the chaotropic salt, centrifuged to separate the silica matrix. The matrix was washed twice with buffer to remove residual chaotrope and polysaccharides, eluted with sterile H_2_O, precipitated with 2 volumes of 95% ethanol, and then dried and resuspended in 100 μL 1 × TE buffer.

### Primer development and PCR amplification

For initial primer design, DNA was extracted from green stem tissue of a wild *S. stellatus* supplied by Huntington Botanical Gardens, San Marino, CA (after removal of the outermost waxy cuticle) using a modified CTAB protocol (Doyle and Doyle 1987) with the addition of caylase. An enriched genomic library was made for the CA/GT motif following the procedure by Kandpal et al. (1994). Repetitive sequences of DNA were identified following the protocols outlined for the cactus *Polaskia chichipe* (Otero-Arnaiz et al. 2004). Primers were designed using the PRIMER3 program (Rozen and Skaletsky 1996; for 6 sequences with microsatellite repeats. Fragments were designed to be between 100 and 300 base pairs in length. The unlabelled primers were tested for amplification and optimized on 18 wild, managed, and cultivated *S. stellatus* individuals collected from the TV and LMB (CoxW, ZapW, SLM, and ChC), as well as four other species of columnar cacti (data not shown). The fragments were separated on 2% agarose gels. Florescent-labeled reverse primers were obtained for five loci (Table 2). MICRO-CHECKER (van Oosterhout et al. 2004) was used to identify genotyping errors; one locus was omitted due to amplification problems.

**Table 2:** Microsatellite loci for *Stenocereus stellatus*

Locus	PCR primer sequence (5′–3′)	Ta (°C)	Size (bp)	n	*A*	mean *A*_O_ (s.e.)	mean *H*_O_ (s.e.)	mean *H*_E_ (s.e.)	mean *H*_O_/mean *H*_E_
JCS1	CCCGAAAGCACATCAAAAAT	52	174–212	494	12	5.33 (0.42)	0.55 (0.05)	0.53 (0.03)	−0.03 (0.06)
	CAGAGAATCGCCAGAGGAAG								
JCS49	CAAACCCAAAAGCAAAGAA	52	192–230	490	19	11.47 (0.95)	0.74 (0.03)	0.81 (0.02)	0.08 (0.04)
	AAGAGACAAGTCCTCAGGTTGG								
JCS51	CCCATGCCAAAATATCAACC	60	200–224	479	9	5.80 (0.51)	0.51 (0.05)	0.52 (0.05)	0.04 (0.04)
	AACTAGGCCCGAAAATGGAT								
JCS68	CATCATTGTCCCACTTAAAGCA	54	195–215	495	13	7.40 (0.62)	0.57 (0.05)	0.63 (0.04)	0.10 (0.05)
	TCCCAAAAACCAAAATCATCA								
JCS73	TGCGAATTAATGGTTTCCAA	54	165–219	483	18	5.47 (0.68)	0.30 (0.04)	0.57 (0.04)	0.49 (0.05)
	TCACATGATGTCATAACAAGCAA								

Locus names, annealing temperature (Ta), size, overall sample size (n), number of alleles (*A*), mean observed number of alleles per population (*A*_O_), mean observed heterozygosity (*H*_O_) and expected heterozygosity (*H*_E_) per population, and the ratio of the last two measures are shown.

For analysis of microsatellites, loci were amplified in 15 μL reactions containing 1 μL genomic DNA (at 5 ng/μL), 0.3 μL primer (at 1 pmol/L), 0.4 μL dNTPs (at 2.5 mmol/L), 0.5 U GoTaq Flexi DNA polymerase (Promega, Madison, WI) and 1.5 μL 10 × GoTaq Flexi amplification buffer (Promega). Annealing temperatures and MgCl_2_ concentrations were independently optimized for each population and each primer (Table 2). Forty cycles of amplification were carried out in an MJ Research Inc. PTC-100 thermalcycler (MJ Research Inc., Waltham, MA), and amplification products were examined on 1.5% agarose gels to verify fragment size. Once conditions were optimized, loci were amplified using fluorescently labeled primers (FAM or HEX) for analysis on an AB3130xl Genetic Analyzer platform (Applied Biosystems, Carlsbad, CA) at RSABG. Amplification products were coloaded with GS-350 ROXsize standard according to manufacturer's specifications. Electrophoretic results were analyzed with GeneMapper software (ver. 4.0; Applied Biosystems, 2005), as well as scored manually.

### Data analysis

Standard measures of genetic diversity within and among populations, *A*_O_ (observed number of alleles), *A*_E_ (effective number of alleles), *H*_O_ (observed heterozygosity), *H*_E_ (expected heterozygosity), and *f* (the fixation index), were calculated using GenAlEx ver. 6.41 (Peakall and Smouse 2006). Bayesian estimates of *f*, *f*_B_, were made using Hickory ver. 1.1 (Holsinger 1999; Holsinger and Wallace 2004) for individual populations and groups of populations (by region, by type, and by region and type), with the advantage of comparing the posterior probability distributions for two samples to determine if estimates were significantly different from one another. Each analysis was performed three times to ensure convergence of estimates. For all Hickory runs, default parameters for burn-in, sample number, and thinning were used. All three models were run and the best one was selected using the deviation information criterion. The traditionally calculated genetic diversity and inbreeding indices were tested for differences among groups with StatView 5.0.1 (SAS Institute, Cary, NC, USA, 2001), with Bonferroni corrections for multiple comparisons.

Analyses of molecular variance (AMOVA) were calculated using GenAlEx ver. 6.41 with 999 total data permutations and pairwise population permutations. Estimates of Nei's genetic distances were made with GenAlEx, and an unweighted pair-group classification based on arithmetic averages (UPGMA) was generated with NTSYSpc 2.2 (Rohlf 2005). Isolation of populations by distance was examined with regression analysis relating *F*_ST_/(1-*F*_ST_) to geographic distance between population pairs. GenAlEx was used to estimate *F*_ST_ and regression parameters, to evaluate model significance with Mantel's tests, and to test for deviation from Hardy–Weinberg expectations. Migration between populations was estimated using BayesAss+ ver. 1.3 (Wilson and Rannala 2003).

BOTTLENECK 1.2.02 (Cornuet and Luikart 1996; Piry et al. 1999) was used to test for deviations from mutation drift equilibrium that may indicate population bottlenecks. All three mutation models (the infinite alleles model, the stepwise mutation model, and the two-phase model [TPM]) were used in preliminary analysis with BOTTLENECK, each with 10,000 iterations; for the TPM, the program was run with a variance of 12 and stepwise contribution of 95% (as recommended by Piry et al. 1999). One-tailed Wilcoxon tests were used to test for a significant excess heterozygosity; Piry et al. 1999 considered this to be the most powerful test for studies with a small number of loci.

To analyze the importance of vegetative propagation in shaping genetic diversity, GenAlEx was used to identify samples with identical multilocus genotypes. For this analysis, individuals with missing data at any locus had to be removed; thus these results may represent an underestimate of the number and size of clones in the populations. To correct for this underestimation, we also identified possible matches for individuals with missing data as other samples that matched at the available loci. The probability of random identity for each repeated genotype was calculated as the product of the allele frequencies over all populations (Peakall and Smouse 2006).

## Results

### Genetic variability

Estimates of genetic diversity at the population level ranged from *H*_E_ = 0.72 in ZapC to *H*_E_ = 0.45 in ZapW, with the second lowest estimate of diversity in EEC (*H*_E_ = 0.48, Table 3). When summarized across the entire study area, mean genetic diversity was greatest in managed populations and lowest in wild populations for all measures examined, but differences were only significant for *A*_O_ and *H*_E_ (Table 4). Cultivated populations showed intermediate values in terms of genetic diversity when considered for both regions collectively. Eight private alleles were detected; all were in either cultivated (4) or managed (4) populations. Private alleles were evenly distributed between the two regions.

**Table 3:** Genetic diversity indices within populations of *Stenocereus stellatus*

Population	*A*_O_ (s.e.)	*A*_E_ (s.e.)	*H*_O_ (s.e.)	*H*_E_ (s.e.)	*f* (s.e.)	*f*_B_ (s.e.)
La Mixteca Baja						
CaC	4.8 (1.32)	3.3 (1.05)	0.46 (0.125)	0.52 (0.154)	0.07 (0.086)	0.165 (0.0306)
CaM	9.0 (1.52)	3.4 (0.90)	0.56 (0.065)	0.65 (0.060)	0.12 (0.080)	0.145 (0.0074)
ChC	9.2 (0.75)	3.8 (0.89)	0.56 (0.088)	0.69 (0.057)	0.21 (0.074)	0.185 (0.0065)
ChM	8.4 (0.81)	3.6 (0.94)	0.52 (0.071)	0.66 (0.059)	0.21 (0.076)	0.187 (0.0080)
ChW	7.0 (1.79)	3.7 (1.31)	0.56 (0.109)	0.62 (0.083)	0.07 (0.145)	0.086 (0.0074)
EEC	4.8 (0.37)	2.0 (0.26)	0.40 (0.093)	0.48 (0.052)	0.22 (0.099)	0.198 (0.0103)
EEM	9.4 (1.57)	4.1 (1.44)	0.55 (0.079)	0.68 (0.060)	0.18 (0.120)	0.192 (0.0116)
EEW	5.8 (1.36)	3.0 (0.82)	0.62 (0.119)	0.59 (0.081)	−0.03 (0.110)	0.053 (0.0075)
Tehuacan valley						
CoxW	7.2 (1.39)	4.0 (0.96)	0.60 (0.110)	0.67 (0.074)	0.14 (0.102)	0.099 (0.0085)
MeC	9.4 (1.75)	3.6 (0.57)	0.47 (0.050)	0.70 (0.037)	0.32 (0.077)	0.310 (0.0067)
MeM	4.2 (0.66)	2.7 (0.29)	0.62 (0.150)	0.61 (0.045)	0.04 (0.204)	0.071 (0.0182)
SJRW	4.8 (1.11)	2.2 (0.36)	0.50 (0.133)	0.51 (0.071)	0.08 (0.203)	0.105 (0.0098)
SLM	8.0 (1.58)	3.4 (0.68)	0.58 (0.131)	0.65 (0.072)	0.16 (0.154)	0.101 (0.0062)
ZapC	9.2 (1.24)	4.2 (1.09)	0.60 (0.042)	0.72 (0.046)	0.15 (0.071)	0.196 (0.0065)
ZapW	6.4 (2.52)	2.4 (0.89)	0.38 (0.096)	0.45 (0.101)	0.15 (0.103)	0.143 (0.0086)

Population abbreviations and diversity statistics are as in Table 2. *A*_E_ is the effective number of alleles; the fixation index (*f*) and a Bayesian estimate of the fixation index (*f*_B_) per population and the standard error of each estimate are presented.

**Table 4:** Measures of genetic diversity by population type and region in *Stenocereus stellatus*

Population group	*A*_O_ (S.E.)	*A*_E_ (S.E.)	*H*_O_ (S.E.)	*H*_E_ (S.E.)	*f* (S.E.)	*f*_B_ (S.E.)
Region and type						
LMB cultivated	7.2 (1.82)^a^	3.1 (0.98)^a^	0.49 (0.116)^a^	0.60 (0.100)^a^	0.20 (0.099)^a^	0.21 (0.004)^a^
LMB managed	8.9 (1.57)^b^	3.7 (1.29)^b^	0.54 (0.084)^b^	0.66 (0.069)^b^	0.17 (0.110)^b^	0.18 (0.002)^a^
LMB wild	6.6 (2.34)^a^	3.4 (1.64)^b^	0.58 (0.160)^b^	0.61 (0.116)^a^	0.05 (0.197)^c^	0.07 (0.005)^b^
TV cultivated	9.3 (2.19)^a^	3.9 (1.25)^a^	0.54 (0.080)^a^	0.71 (0.060)^a^	0.23 (0.120)^a^	0.26 (0.003)^a^
TV managed	7.3 (2.31)^b^	3.2 (0.90)^a,b^	0.59 (0.190)^a^	0.64 (0.095)^b^	0.13 (0.234)^b^	0.08 (0.005)^b^
TV wild	6.3 (2.26)^c^	2.9 (1.05)^b^	0.48 (0.139)^b^	0.54 (0.116)^c^	0.13 (0.158)^b^	0.17 (0.003)^a,b^
Region						
LMB	6.9 (1.07)	3.3 (0.72)	0.53 (0.070)	0.61 (0.056)	0.14 (0.078)	0.178 (0.001)
TV	6.4 (1.27)	3.2 (0.67)	0.53 (0.091)	0.60 (0.070)	0.13 (0.117)	0.20 (0.001)
Population Type						
Cultivated	6.5 (1.27)^a,b^	3.3 (0.79)	0.51 (0.088)	0.63 (0.069)^a,b^	0.18 (0.102)^a^	0.24 (0.002)^a^
Managed	7.4 (1.42)^a^	3.4 (0.88)	0.56 (0.962)	0.64 (0.066)^a^	0.13 (0.127)^a,b^	0.16 (0.002)^b^
Wild	6.0 (1.53)^b^	3.0 (0.90)	0.51 (0.110)	0.55 (0.091)^b^	0.08 (0.124)^b^	0.14 (0.002)^b^

Weighted means to correct for unequal sample sizes are presented. Superscripts indicate significantly different measures. For the region and type population group, only the comparisons within regions are shown here (other comparisons are discussed in the text). For population abbreviations and diversity statistics refer to Table 1.

Populations in the two regions did not differ significantly overall in mean genetic diversity or levels of inbreeding (Table 4). Several significant contrasts were evident, however, among means for genetic diversity for cultivated, managed, and wild populations within each region, the specific pattern of which differed between the two regions (Table 4). In LMB, cultivated populations had lower estimates of genetic diversity and higher estimates of inbreeding, whereas managed populations typically had the highest diversity and intermediate levels of inbreeding. In the TV, wild populations had the lowest measures of genetic diversity and cultivated populations, the highest; in fact, values for all four genetic diversity measures differed significantly between these two management groups. The managed and wild population types showed two significant differences in diversity, *A*_O_ (*P* = 0.036) and *H*_E_ (*P* = 0.011), with managed populations having greater diversity in both cases. The cultivated and wild population types showed a significant difference in the fixation index, *f* (*P* = 0.007), with wild populations having less inbreeding than cultivated ones.

Bayesian estimates of the fixation index, *f*_B_, were similar to estimates of more traditionally calculated methods (*f,* Tables 4). Among population types overall, *f*_B_ was significantly higher in cultivated populations than in either managed or wild populations, but managed and wild populations did not differ significantly in *f*_B_. There was no significant difference between LMB and the TV at the regional level for either measure.

There was a trend for more deviations from Hardy–Weinberg expectations for individual loci in the cultivated populations, then the managed populations, followed by the wild populations (66.7%, 52.0%, 40.0%, respectively), but these differences were not statistically significant (*P* = 0.17). There was also no statistically significant difference between the TV and LMB (54.3% vs. 53.9%, respectively, *P* = 0.98).

Populations showed little evidence of bottlenecks, regardless of the mutation model used. The only population with a significant excess heterozygosity based on a one-tailed Wilcoxon test was CoxW (*P* = 0.047) under the infinite alleles model; no population showed a significant departure in the appropriate direction from mutation drift equilibrium under the other mutation models tested. Many populations were characterized instead by a heterozygosity deficiency. With 60 separate tests, we would expect three to be significant by chance based on a probability level of 0.05; therefore the significant result for CoxW may or may not be meaningful and is being examined further as part of a separate analysis (Cruse-Sanders, unpublished data).

### Genetic structure

The AMOVA indicated that significant genetic variation was evident both among populations and between regions, but not among management categories. For all populations, 93% of the variation was found within populations, 6% among populations, and 1% between regions (all *P* < 0.001). At the sub-regional scale, twice as much genetic variation was evident among populations in the TV (8%) as in LMB (4%, both *P* < 0.001). Within both regions, all remaining variation was maintained within populations (92% and 96%, respectively), with no variation among management categories in either case. The mean value for Nei's genetic distance was greater in the TV than in LMB (*D* = 0.140 ± 0.015 SE vs. 0.102 ± 0.105, respectively). The three managed populations examined in LMB had the lowest mean *D* value to all other populations, but in the TV, managed populations had two of the greatest mean *D* values.

**Figure 3:**
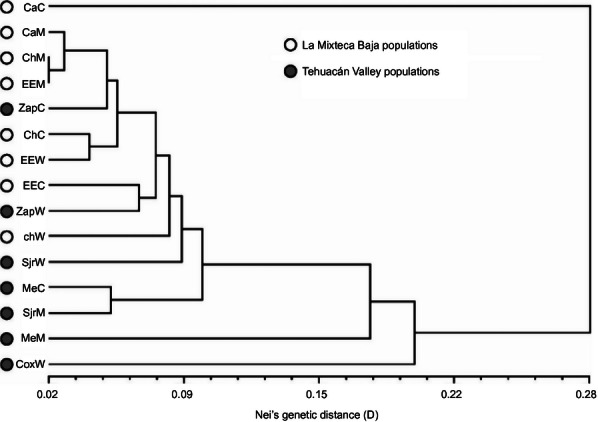
Phenogram representing UPGMA generated with NTSYSpc 2.2 based on estimates of Nei's genetic distances. Symbols represent two different regions, the TV and LMB. Refer to text and Table 1 for population names that correspond to abbreviations in the phenogram. UPGMA, unweighted pair-group classification based on arithmetic averages; TV, Tehuacán Valley; LMB, La Mixteca Baja region.

The patterns of genetic similarity depicted in the UPGMA show a general separation of LMB and TV populations (Fig. 3). CaC, CoxW, and MeM were outliers. Most of the LMB populations joined together at relatively low levels of genetic distance (eg, CaM, ChM, and EEM). The pattern was less cohesive for the TV, with the two Zapotitlan populations (ZapC and ZapW) joining with LMB populations before other populations in their own region. Despite the separation of the two regions on the UPGMA, no significant isolation by distance was apparent for either the study area overall or separately within each region.

Bayesian assignment analyses, as implemented in BayesAss+, estimates the probability of the population of origin for an individual, and the probability of migration of a given individual from a given population into a given population. Non-zero estimates of assignment between two different populations can indicate migration or gene flow between those two populations. This analysis has the advantage of being able to estimate unidirectional gene flow between populations. In only two cases were estimates of migration rate between populations in different regions significantly greater than zero. These were from both SjrW and ZapC in the TV to CaM in LMB.

As hypothesized, there were more significant estimates of migration rate within LMB than within the TV (8 vs. 3; Tables 6), which is not surprising in view of the tighter clusters on the UPGMA and the smaller mean genetic distance D value. Within LMB, most of the significant migration was from CaM; in fact, migration from CaM to all other populations but CaC was significantly greater than zero. The other population that was a source of significant migration was also a managed population, from ChM to both CaC and ChW. CaM was the most centrally located population in this region, with the lowest mean geographic distance to other populations. In the TV, the only source of intraregional migration was MeC; significant estimates of migration occurred from that population to the two geographically closest populations (MeM and ZapC) and one of the more distant populations, SLM. All thirteen significant estimates but one were of migration originating in either cultivated or managed populations. Although some of the larger populations were sources of migrants, a relationship among population size, density, and migration was not clearly expressed. Not all large populations served as sources of migrants; some instead received migrants. One source of migrants in LMB (CaM) had the lowest density of the populations studied (see Table 1).

**Table 5:** Estimates of the mean probability of assignment of an individual between pairs of populations of *Stenocereus stellatus* in La Mixteca Baja (mean±s.d.)

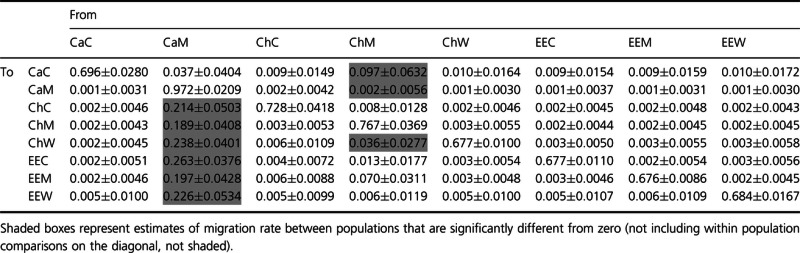

**Table 6:** Estimates of the mean probability of assignment of an individual between pairs of populations of of *Stenocereus stellatus* in Tehuacán VValley (mean±s.d.)

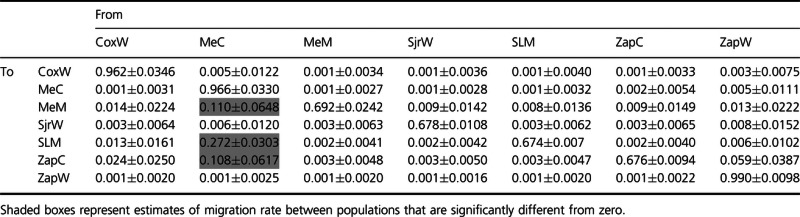

### Clonal structure

Our analysis of clonal structure found 14 genotypes that were shared either within or among populations of *S. stellatus*. In most populations, each individual sampled had a different genotype, although in some of those cases genotypes were shared with another population (Table 7). EEC had the highest occurrence of duplicated genotypes, with only 77% of genotypes unique. Many repeated genotypes (8 of 14) were present in only two sampled individuals and most (11 of 14) represented within-population duplication. Four genotypes were shared between two populations, H, I, K and N, with two of these shared between EEC and ChC. The probabilities of random identity (ie, the probability that two individuals share a genotype by chance rather than by clonal relationships) varied greatly among genotypes, from 1.10 × 10^−8^ to 0.168 (Table 8). Therefore, it is possible that some of the shared genotypes represent chance identity rather than a clonal relationship. In LMB, genotypes were shared within only cultivated populations. In contrast, all genotypes in cultivated TV populations were unique; and repetition of genotypes was evident within both of the managed populations examined, as well as two of the three wild populations.

**Table 7:** Population sample sizes, number and percentage of unique genotypes (indicated by different letters) within *Stenocereus stellatus*, and shared genotypes within and among populations

Population abbreviation	Sample size	Number of unique genotypes	% unique genotypes	Shared genotypes within populations	Shared genotypes among populations
La Mixteca Baja					
CaC	10	10	100		
CaM	34	34	100		H
ChC	41	40	98	A,	I, K
ChM	36	36	100		
ChW	40	40	100		N
EEC	31	24	77	J, L	I, K
EEM	40	40	100		N
EEW	16	16	100		
Tehuacán valley					
CoxW	32	32	100		
MeC	42	42	100		
MeM	10	8	80	O	
SjrW	27	25	93	B, E	
SLM	40	36	90	C, G	
ZapC	40	40	100		
ZapW	42	36	86	D, F, M	H

**Table 8:** Shared genotypes within and among populations of *Stenocereus stellatus*, the populations in which they occur, and the probability of random identity for each population

Genotype	Population(s) in which it is observed	Number of occurrences	Probabilities of random identity
A	ChC	2	1.10×10^−8^
B	SjrW	2	1.04×10^−3^
C	SLM	2	4.92×10^−5^
D	ZapW	2	4.06×10^−4^
E	SjrW	2	1.81×10^−4^
F	ZapW	2	2.40×10^−3^
G	SLM	4	1.10×10^−4^
H	ZapW	2	0.017
	CaM	1	1.22×10^−3^
I	EEC	3	9.90×10^−4^
	ChC	1	2.20×10^−6^
J	EEC	2	6.98×10^−3^
K	EEC	2	5.71×10^−3^
	ChC	1	6.04×10^−5^
L	EEC	4	4.78×10^−3^
M	ZapW	2	1.03×10^−4^
N	EEM	1	7.20×10^−7^
	ChW	1	4.39×10^−5^
O	MeM	3	4.91×10^−5^

## Discussion

This study is the first to provide detailed analysis of population processes within the long-lived, semi-domesticated species, *Stenocereus stellatus*. It also provides a node for comparative evolutionary studies of plants that are important for traditional agriculture in marginal, arid, or tropical environments. The pattern of diversity seen in *S. stellatus* differs from the classical model of ex situ domestication associated with narrowing of genetic diversity existing wild crop relatives through artificial selection (Hawkes 1983; Doebley 1992; Hollingsworth et al. 2005), but it supports more recent findings of enhanced genetic diversity in some cases of in situ domestication in traditional agricultural systems in Mesoamerica (Otero-Arnaiz et al. 2005b; Zizumbo-Villarreal et al. 2005; Casas et al. 2007; Parra et al. 2008, 2010). Our comparisons of genetic diversity, inbreeding, bottleneck occurrence, gene flow, and genetic structure of populations grown under different management systems in LMB and TV help elucidate the processes that are part of in situ domestication. They demonstrate that cultivation of plants in close proximity to wild populations leads to a complex interaction of natural processes and cultural practices that collectively shape patterns of genetic variation across the landscape.

### Genetic diversity and inbreeding

We found greater microsatellite diversity in *S. stellatus* populations that were under at least moderate human manipulation than in wild populations, as we had hypothesized. Private alleles were only evident in cultivated and managed populations, most likely the result of farmers introducing novel stock from other gardens, markets, or more remote wild populations. The specific pattern of genetic diversity among the three groups differed somewhat between the LMB and TV regions. With respect to broad patterns of genetic diversity, our findings generally paralleled those reported previously with allozyme-based analyses for this species (Casas et al. 2006), with some differences. The effective number of alleles in cultivated populations of the TV was significantly higher than in managed or wild populations, and the effective number of alleles in managed populations was intermediate and not significantly different than in wild populations. The same was true for the pattern of expected heterozygosity within populations of the TV, however, with significantly lower diversity in wild populations. Among manipulated populations sampled from LMB, managed populations had significantly higher expected heterozygosity and higher effective number of alleles compared with cultivated or wild populations. This may reflect more active management in LMB that goes along with more commerce in *S. stellatus* in this region as noted by interview data. This study contributes new information about the mechanisms by which higher levels of genetic diversity are maintained in human-manipulated populations and the interplay between cultivation practices and ongoing natural processes of selection and pollinator-mediated gene flow.

As with columnar cacti, studies of incipient domestication in tropical and subtropical trees tell a complicated story of how management impacts evolutionary processes and genetic diversity within these species. Several African taxa, including *Vitex fischeri* Gürke. and *Vitellaria paradoxa* C.F.Gaertn., showed similar levels of genetic diversity in wild and planted populations (Kelly et al. 2004; Lengkeek et al. 2006), whereas diversity in *Inga edulis* Mart. was significantly lower in planted than wild populations in Peru (Hollingsworth et al. 2005). Miller and Schaal (2006) reported that *Spondias purpurea* L. orchards in Mexico had lower genetic diversity than wild populations, but home gardens did not, which they attributed to the typical practice of planting only one or two varieties in orchards, but many varieties in home gardens. Contrasts in these findings have been attributed to differences in cultivation history, intensity of human management, proximity of wild and cultivated populations, and generation time of the species (Hollingsworth et al. 2005; Lengkeek et al. 2006). Like *S. stellatus*, *Spondias purpurea* in home gardens is likely managed for phenotypic fruit diversity (eg, different colors, tastes [Brush et al. 1995]) and may have even stronger selection for diversity than in the other tropical trees.

Despite their greater genetic diversity, cultivated *S. stellatus* populations showed a greater degree of inbreeding than either in situ managed or wild populations. Considering the self-incompatible breeding system (Casas et al. 1999b) and high motility of the bat pollinators, we would not expect inbreeding estimates for wild *S. stellatus* populations to differ significantly from zero. Parra et al. (2010) also found positive inbreeding coefficients for managed populations of the congeneric cactus, *Stenocereus pruinosus*, managed in the same region of Mexico, however, overall inbreeding values were not significantly different than zero.

Several possible explanations may account for the inbreeding observed. First, significant estimates of *f* may have resulted from the Wahlund effect in cultivated populations, potentially a consequence of pooling home garden samples. Differences in allele frequencies among gardens could generate significantly positive estimates of *f* without inbreeding. Second, plants in cultivation are often more inbred, as individuals with recessive mutations may be under positive human selection, reinforced through asexual recruitment. This explanation is in keeping with the greater morphological variation in cultivated populations, particularly in terms of fruit color (Casas et al. 2006). If *S. stellatus* is an obligately outcrossing species, rare naturally occurring or human-induced inbred individuals likely exhibit mutant phenotypes because of their high genetic load (Wiens et al. 1987). Fruit color diversity in cultivated populations probably represents loss-of-function mutations and thus are likely genetically recessive. Both positive (Perales et al. 2005) and negative (Pujol et al. 2005; Parker et al. 2007) estimates of *f* have been reported previously.

### Importance of asexual recruitment

We had expected differences among management groups in the prevalence of asexual recruitment, with subtle regional contrasts due to differences in the physical and cultural environments. Populations conformed to our expectations in LMB, where multilocus genotypes were repeated within two of the three cultivated but no other populations. Probabilities of random identity for repeated genotypes were sufficiently low that genotype duplication likely resulted from planting of identical stem pieces. In the TV, however, populations showed nearly the opposite pattern. Multilocus genotypes were not repeated within any cultivated population, but they were within two of the three wild and both managed populations examined.

Different forces that affect the balance between sexual and asexual recruitment have likely been at play within each region. In cultivated populations of LMB, asexual recruitment was likely associated with management practices aimed at introducing desired phenotypic traits into gardens. In the TV, where more arid conditions limit the frequency of sexual recruitment, the occurrence of vegetative propagation in managed and wild populations likely reflects limitations imposed by the physical environment more than cultivation practices. Casas et al. (1999a) reported that *S. stellatus* seedlings were common in LMB, but rare in the much drier TV. Seed germination and seedling success depend on humidity (Rojas-Arechiga et al. 2001); and field experiments indicate that seeds germinate during unusually wet years in the TV, yet fail to establish (Casas et al. 2002). The lower levels of genetic diversity observed in wild TV populations relative to LMB may have resulted from lower sexual recruitment in this region, and the consequent reduced gene flow, despite the potential of bats to move pollen long distances. The absence of repeated multilocus genotypes within cultivated populations of the TV (particularly when they were present in LMB) was surprising and may reflect the spacing of potentially repeated genotypes within the specific gardens sampled.

Future water availability is predicted to decrease with changing climate in the Tehuacán Valley. With increasing human population size, drought, and pollution, cactus populations may become smaller or more fragmented across the landscape. Interview data indicated that *S. stellatus* continues to provide an important source of fruit in a region with limitations for growing traditional crops; therefore, management and population patterns in this species will likely continue evolving with changing climate in the future.

### Evidence for bottlenecks

Many cultivated species have gone through a bottleneck during domestication resulting from founder effects and continued human selection (Doebley 1989). In the case of *S. stellatus*, however, neither in situ managed nor cultivated populations showed evidence of bottlenecks. Many of the populations exhibited a heterozygosity deficit relative to expectations under mutation drift equilibrium, instead of the excess that is indicative of past bottlenecks. Cornuet and Luikart (1996) emphasized that the heterozygosity excess associated with the detection of a bottleneck is different from an excess of heterozygotes. In the former case, the observed heterozygosity is compared with expectations based on the observed number of alleles, given mutation-drift equilibrium, whereas in the latter case the number of heterozygotes is compared with Hardy–Weinberg equilibrium expectations.

Several factors may account for the absence of bottlenecks in the *S. stellatus* populations examined. When bottlenecks occur during domestication, the decrease in effective population size associated with artificial selection causes allelic richness and heterozygosity to decline (Cornuet and Luikart 1996), with the former decreasing more rapidly. As a bottleneck develops, the allele deficiency increases to a peak, then falls to zero again until mutation-drift equilibrium is re-established through mutation or immigration. The duration and magnitude of the peak in allele deficiency depends on effective population size before and after the bottleneck initiation, time since its onset, generation time of the species, and rates of mutation and introduction of new alleles through either introgression with wild plants or planting by farmers (Eyre-Walker et al. 1998). Bottlenecks associated with domestication may be as short as several centuries (Diamond 1997) but may last millennia (Eyre-Walker et al. 1998). In the case of *S. stellatus*, management practices may have helped the species escape a bottleneck. According to archeological information (Callen 1965), *S. stellatus* has been used by humans for more than 5000 years, and this species grows relatively rapidly, with generation times shortened by clonally propagated plants (Casas et al. 2002). However, *S. stellatus* may not have been the focus of intense management until recently; hence insufficient time may have passed for a domestication bottleneck to be expressed, particularly given the longevity of the species.

In addition to these substantive influences, several artifacts of the data may have influenced the BOTTLENECK analysis. The program's power to identify bottlenecks depends on the number of loci; whether populations have substructure, immigration, or departures from Hardy–Weinberg equilibrium (eg, inbreeding); the mutation model; and the statistical test used to detect heterozygosity excess (Cornuet and Luikart 1996). Although our samples met the minimum requirement of four loci for BOTTLENECK, the power of the analysis to detect bottlenecks increases with additional loci (Cornuet and Luikart 1996; Piry et al. 1999). Substructure and recent immigration within populations can counter the loss of alleles that occurs in bottlenecking, mimicking postbottleneck population expansion, and thereby hindering bottleneck detection. Domestication in many crop species, including *S. stellatus*, appears to be a protracted process with continuing natural gene flow between wild and manipulated populations for many years, as well as human-mediated gene flow associated with the manipulation and exchange of plant stock (Allaby et al. 2008). Management aimed specifically at increasing diversity may offset selection pressures that would otherwise reduce diversity, thereby slowing or preventing occurrence of bottlenecks.

### Gene flow and patterns of similarity among populations

The vast majority of significant estimates of gene movement in our analysis represent within-population matings, similar to the findings of Otero-Arnaiz et al. (2005a). This effect is expected in a cactus species like *P. chichipe* with diurnal flowers pollinated by generalist bees; however, it is unexpected for a self-incompatible species like *S. stellatus* with a bat pollination syndrome and underscores the effects of human manipulation. Very little gene flow has apparently occurred between regions, despite the possibility of bat flight over those geographic distances.

The phenogram depicting population similarities showed a division between the two regions, with details of some population clusters conforming to expectations and details of others countering them. Populations grouped more cohesively in LMB than in the TV. The lower intraregional gene flow in the TV relative to LMB has resulted in stronger differentiation among populations, as indicated by the AMOVA results and greater mean genetic distance to other populations within the TV. Interviews revealed that farmers in LMB frequently planted seed from fruits obtained in regional markets in addition to stem pieces in their gardens, in contrast to the TV where planting was primarily by vegetative propagation. Two interviewees in LMB reported traveling some distance to obtain stock for their gardens, which would result in greater gene flow and blurring of genetic distinctions among populations. The asexual recruitment that occurred in most wild and managed populations of the TV (but not LMB) would reinforce existing differentiation among populations, as has been reported for other species characterized by asexual recruitment.

At the intraregional spatial scale, populations did not show a clear geographic pattern of similarity between wild and nearby human manipulated populations, and isolation by distance was not evident. Within regions, migrants only originated in cultivated and managed populations, not wild populations. Some gene flow was from a centrally located population to nearby populations, but in other cases, gene flow occurred between some of the more distant populations. We detected no clear relationship between population size or density and gene flow.

As is occurring elsewhere in Mexico (eg, Zizumbo-Villarreal et al. 2005), the study region is undergoing transitions in land ownership and composition of the workforce, which both affect manipulation of *S. stellatus* in managed and cultivated populations. Although managed populations have historically been primarily on communal land accessible to all town residents, part of one managed population we visited (CaM) had recently been developed, with conversion to home gardens as private residences filled in the population. Another (MeM) is now under the ownership of several town residents who have augmented the managed population with additional stem pieces to curb soil erosion and supply fuel for pottery firing (Cruse-Sanders and Parker, interview data). Patterns of gene flow and genetic similarity involve human agency where there are close cultural ties between the populations and farmers move stem pieces or seeds from one population to another. They may also reflect pollinator-mediated gene movement, as the distance between many of our populations is within the range that bats may move in a night of foraging. Superimposed on these more predictable processes are the idiosyncrasies of individual towns and farmers pertaining to land ownership and goals of management, interactions with local and regional markets, and individual preferences for different fruit traits. Together they constitute a complex web of cultural, physical, and biological interactions that have shaped the composition of the populations we examined.

### Implications for our understanding of domestication

This analysis of *S. stellatus* joins a growing literature that increases our appreciation of the complexities of domestication in traditional agricultural systems, particularly crops adapted to arid environments in an age human mediated of global change. Rather than following a uniform model of reduced genetic variation and bottlenecking, domestication in traditional systems plays out differently for different species, with strong (and often opposing) selective pressures imposed by both agricultural and natural environments (Allaby et al. 2008). In some species (eg, *S. stellatus, S. pruinosis* [Parra et al. 2010; ]), farmers select plants to maximize diversity within home gardens as a form of bet-hedging to ensure consistent production under variable climatic conditions and to maintain reliable crop sources for a variety of uses (Brush et al. 1995; Pujol et al. 2005; Zizumbo-Villarreal et al. 2005; Parra et al. 2010). In these cases, domestication is a complicated and protracted process that involves artificial selection in managed populations and home gardens, gene exchange between wild and cultivated populations effected by both native pollinators and human interactions, and natural selection in varying environments. In such species, manipulated populations play a significant role in the patterning of diversity across the landscape. Intense management practices and ongoing natural gene flow in these species counteracts the loss of diversity associated with initial founder effects, thus preventing or slowing genetic bottlenecks reported for many crops (Doebley 1989; Eyre-Walker et al. 1998), which are often considered an integral component of domestication.

Since the mid-20th century, growing concerns about conservation of crop genetic resources and increasing alarm over food security, especially in areas of low agricultural productivity, have resulted in incorporation of these issues into national policies and international agreements (Gepts 2008; Brown 2011). As molecular markers become available for nontraditional crops and novel analysis techniques allow us to detect and evaluate patterns of genetic diversity, this type of research is essential for understanding evolutionary processes within species manipulated by humans. Indeed, they can provide valuable insights into managing crop genetic resources into the future against a backdrop of climate change, and they underscore the importance of traditional agriculture systems in maintaining genetic diversity for plant species.
